# Relevance of Brain Lesion Location to Cognition in Relapsing Multiple Sclerosis

**DOI:** 10.1371/journal.pone.0044826

**Published:** 2012-11-05

**Authors:** Francesca Rossi, Antonio Giorgio, Marco Battaglini, Maria Laura Stromillo, Emilio Portaccio, Benedetta Goretti, Antonio Federico, Bahia Hakiki, Maria Pia Amato, Nicola De Stefano

**Affiliations:** 1 Department of Neurological and Behavioral Sciences, University of Siena, Siena, Italy; 2 Department of Neurology, University of Florence, Florence, Italy; University Hospital La Paz, Spain

## Abstract

**Objective:**

To assess the relationship between cognition and brain white matter (WM) lesion distribution and frequency in patients with relapsing-remitting multiple sclerosis (RR MS).

**Methods:**

MRI-based T2 lesion probability map (LPM) was used to assess the relevance of brain lesion location for cognitive impairment in a group of 142 consecutive patients with RRMS. Significance of voxelwise analyses was p<0.05, cluster-corrected for multiple comparisons. The Rao Brief Repeatable Battery was administered at the time of brain MRI to categorize the MS population into cognitively preserved (CP) and cognitively impaired (CI).

**Results:**

Out of 142 RRMS, 106 were classified as CP and 36 as CI. Although the CI group had greater WM lesion volume than the CP group (p = 0.001), T2 lesions tended to be less widespread across the WM. The peak of lesion frequency was almost twice higher in CI (61% in the forceps major) than in CP patients (37% in the posterior corona radiata). The voxelwise analysis confirmed that lesion frequency was higher in CI than in CP patients with significant bilateral clusters in the forceps major and in the splenium of the corpus callosum (p<0.05, corrected). Low scores of the Symbol Digit Modalities Test correlated with higher lesion frequency in these WM regions.

**Conclusions:**

Overall these results suggest that in MS patients, areas relevant for cognition lie mostly in the commissural fiber tracts. This supports the notion of a functional (multiple) disconnection between grey matter structures, secondary to damage located in specific WM areas, as one of the most important mechanisms leading to cognitive impairment in MS.

## Introduction

Cognitive impairment occurs in a significant proportion of patients with multiple sclerosis (MS) [1], [2]. It tends to worsen with time, but might be relevant even at the earliest disease stages and is primarily characterized by reduced information processing speed, visual learning, working memory, long-term memory and executive functioning [1]–[3]. Given the high occurrence of cognitive impairment in MS and its impact on the patients' daily life, an accurate definition and understanding of the mechanisms underlying its development is essential.

Since magnetic resonance imaging (MRI) is the most sensitive tool to investigate *in vivo* tissue damage occurring in the MS brains, many recent studies have used quantitative MRI-based techniques to improve the knowledge of the substrates underlying cognitive impairment in MS [4]. Indeed, several studies have shown that greater lesion burden and brain atrophy occur in MS patients with a more severe cognitive impairment [5]–[15]. In particular, the pathologic substrate of cognitive impairment seems to be closely related with grey matter (GM) damage such as cortical atrophy [11], [13], [16] and cortical lesions [17], [18], while this relationship is less certain with macroscopic white matter (WM) damage. In such a context, however, few recent studies have supported the early notion that fiber disconnection, caused by both macroscopic and microscopic WM damage, may represent one of the main factors contributing to the development of cognitive impairment in MS [19]–[21].

Among different MRI-derived approaches, lesion mapping has shown to be a very powerful tool for studying distribution and probability of occurrence of brain lesions [22]–[35], thus providing clinically relevant information. In MS patients with cognitive impairment, this should allow the visualization of spatial patterns of focal pathology that would appear much less evident in single patient studies, potentially providing precious information on domain-specific disconnection phenomena due to WM lesions. Thus, with the aim to ascertain the relevance for cognition of WM lesion spatial distribution and frequency, we created lesion probability maps (LPMs) in two groups of relapsing remitting (RR) MS patients with and without cognitive impairment.

## Methods

### Ethics statement

Informed written consent was obtained from all subjects and the study was approved from the ethics committees of the University of Siena and University of Florence.

### Patient population

We studied 142 patients (107/35 women/men; age: mean 39.4 years, range 18.5–57.5 years) with clinically definite MS and RR course according with the revised McDonald criteria [36]. Patients were recruited among those who were consecutively referring to the MS Clinics of the Universities of Siena and Florence during the study period. All the patients were relapse-free and were not taking steroids for at least one month prior to the assessment. No subject was taking psychoactive drugs or substances that might interfere with neuropsychological performance. Seventy-eight out of 142 patients were on treatment (74 with immunomodulatory and four with immunosuppressive drugs). All the patients underwent a neurological evaluation, which included assessment of the Expanded Disability Status Scale (EDSS) [37], neuropsychological evaluation and conventional MRI scans within one-week interval.

### Neuropsychological assessment

In each center, neuropsychological testing was performed by a well-trained psychologist who had participated in a common training session and was blinded to the MRI results. Cognitive functioning was measured through the Rao Brief Repeatable Battery (BRB) of Neuropsychological Tests [38], which incorporates tests of verbal memory acquisition and delayed recall (Selective Reminding Test – SRT), visual memory acquisition and delayed recall (10/36 Spatial Recall Test – SPART), attention, concentration and speed of information processing (Paced Auditory Serial Addition Test – PASAT; Symbol Digit Modalities Test – SDMT) and verbal fluency on semantic stimulus (Word List Generation -WLG). Neuropsychological performance was assessed using the normative data for the Italian population [39]. In particular, failure of a test was defined as a score at least 2 SDs below the mean normative values. Patients with significant cognitive impairment were defined as those showing the failure on at least two tests of the BRB.

### MR examination

All patients were examined using an identical MR protocol. Acquisitions of brain MRIs were obtained in a single session using a Philips Gyroscan at 1.5 T (Philips Medical Systems, Best, The Netherlands). A sagittal survey image was used to identify the anterior and posterior commissures (AC and PC). A dual-echo, turbo spin-echo sequence (TR/TE1/TE2  =  2075/30/90 ms, 256×256 matrix, 1 signal average, 250 mm field of view [FOV], 50 contiguous 3 mm slices) yielding proton density (PD) and T2-weighted (T2-W) images was acquired in the axial plane parallel to the AC-PC line. Subsequently, axial T1-weighted (T1-W) gradient echo images (TR/TE = 35/10 milliseconds, 256×256 matrix, 1 signal average, 250×250 mm field-of-view) were acquired. This sequence yielded image volumes of 50 slices, 3-mm thick, oriented to match exactly the PD/T2 acquisition.

Periodical quality assurance sessions and no major hardware upgrades were carried out on the scanner during the time of the study.

### Lesion volume

For each RRMS patient, MRI scan was first visually assessed by a single observer, unaware of subject identity, and then labeling of T2 lesions was performed by employing a semiautomated segmentation technique based on user supervised local thresholding (Jim 5.0, Xinapse System, Leicester, UK). For the T2-lesion classification, lesion borders were determined primarily on PD images but information from T2-W images was also considered. The values of total brain T2-lesion volume (LV) was calculated by multiplying lesion area by slice thickness.

### Lesion probability maps

For each MS group, LPMs were obtained by using imaging analysis tools of the FMRIB Software Library (FSL (www.fmrib.ox.ac.uk/fsl/) [40] as described here:

1. The brain was extracted from the T1-W and PD images using BET and then corrected for intensity nonuniformity [41]. The same number of T1-W images of both cognitively impaired (CI) and cognitively preserved (CP) patients were randomly chosen from the total population and linearly registered to the MNI152 standard brain by using FLIRT. Then, by averaging the registered images, a symmetric study-specific template was obtained [27].
2. A two-stage registration was carried out to align the T2-lesion mask of each patient to the template. First, each lesion mask was linearly registered on the T1-W image using the transformation parameters derived by linearly registering the PD on the T1-W image. Second, each lesion mask previously registered on the T1-W image was nonlinearly registered on the template with FNIRT using the transformation parameters derived by nonlinearly registering the T1-W image on the template. The nearest neighbor interpolation method was used in both stages. Quality checks were done at all steps by two independent observers (F.R. and M.B.), who found the registration to be satisfactory in all cases.
3. Finally, for each patient group, LPM was generated by first merging and then averaging all the standard-space lesion masks. For each map, voxel intensity represents the frequency of lesion occurrence in that voxel or, in other words, the probability of that voxel being lesion for the patient group.
4. As the last step, we computed a measure of the consistency of lesion pattern across patients of each group. This was described as LPM index (LPMi) in a previous work [22] and was obtained by performing the equation

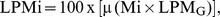
in which Mi is the binarized, segmented lesion mask of the patient, LPM_G_ is the LPM of the corresponding patient group and μ is the resulting mean. As a result of this procedure, LPMi is higher when the patient's lesion mask is localized in brain regions with a high probability of being lesion for that group.

### General statistics

Nonparametric Mann-Whitney test was used for between-group comparisons of demographic (age), clinical (EDSS, disease duration) and MRI (T2-LV and LPMi) features. To account for differences in head size, for each patient LV was measured in common standard space, and this was used in all subsequent analyses. The Fisher's exact test was used to compare sex between the two groups of CI and CP patients. Data were considered significant at p<0.05.

**Table 1:** Demographic, clinical and MR data of the MS population.

	Total	CP group (n = 106)	CI group (n = 36)
Age, y, mean±SD	39.4±9.1	38.2±8.8	42.8±9.12
(range)	(18.5–57.5)	(18.5–57.5)	(20.6–56.6)
Male/Female	35/107	26/80	9/27
EDSS, mean±SD	1.8±1.2	1.7±1.1	2.2±1.4
(range)	(0–6)	(0–5.5)	(0–6)
Disease duration, y,	11.0±9.8	11.2±10	10.6±9.1
mean±SD (range)	(0.02–41.6)	(0.02–41.6)	(0.3–35.2)
T2-LV, cm^3^, mean±SD	13.1±13.5	11.2±12.6	18.8±14.4
(range)	(0.1–60.2)	(0.1–60.2)	(0.2–44.4)

CP =  Cognitively Preserved.

CI = Cognitively Impaired.

See text for details.

### Voxelwise statistics

Voxelwise statistical inference on LPM was done in the General Linear Model (GLM) framework with *randomise*, an FSL program, which performs nonparametric permutation testing (5000 permutations) [42]. To compare probability of lesion occurrence between the two patient groups (CP and CI) a voxelwise unpaired t-test was used. To correlate probability of lesion occurrence with the scores of the different tests of BRB across the whole patient population voxelwise regression analyses were used. In all analyses, age and sex were used as covariates. In addition, since it is not clear whether the total brain LV can be relevant to lesion frequency assessment [23], [25], [26], all analyses were repeated adding T2-LV as a covariate. Values of T2-LV were log-transformed in order to obtain a normal distribution. Further, an analysis on three groups was performed to assess the relevance of the number of failed tests. At F-contrast testing among these three groups, the main effect of group (i.e., showing the brain regions with significant heterogeneity) was created and used to mask all subsequent pairwise comparisons.

Thresholding was carried out using TFCE (Threshold-Free Cluster Enhancement), a method for finding significant clusters in MRI data without having to define them in a binary way [43]. Clusters were considered significant at p<0.05, fully corrected for multiple comparisons across space. Within clusters showing significant correlation of lesion frequency with BRB test scores, we extracted for each patient the mean signal intensity value (i.e., the percentage of lesion voxel) across the voxels within the significant cluster and correlated this with BRB test score in order to assess the Spearman correlation coefficient between the two parameters.

Anatomical location of the significant clusters was determined by using the Johns Hopkins University Diffusion Tensor Imaging-based atlases of WM anatomy provided by FSL.

## Results

### Demographic and clinical characteristics

Demographic, clinical and MRI data of our population are summarized in Table 1. The BRB identified 36 CI patients, while 106 patients were CP. Number of patients failing each test of the BRB is shown in Table 2. CI patients were older than CP patients (p = 0.008) and tended to be more disabled (p = 0.06), but no differences were found in disease duration (p = 0.7). T2-LV was significantly higher in CI than in CP patients (p = 0.001).

**Table 2:** Neuropsychological tests failed by cognitively impaired MS patients.

Neuropsychological test	No. of patients
SDMT	17/36
PASAT	16/36
SRT-LTS	16/36
SRT-CLTR	16/36
SPART	13/36
SPART-DR	8/36
WLG	8/36
SRT-DR	7/36

SDMT  =  Symbol Digit Modalities Test.

PASAT =  Paced Auditory Serial Addition Test.

SRT-LTS =  Selective Reminding Test- Long Term Storage.

SRT-CLTR =  Selective Reminding Test Consistent- Long Term Retrieval.

SPART =  Spatial Recall Test.

SPART-DR =  Spatial Recall Test- Delayed Recall.

WLG =  Word List Generation.

SRT-DR =  Selective Reminding Test -Delayed Recall.

### LPM Analyses

#### Comparison between CP and CI groups

**Figure 1:**
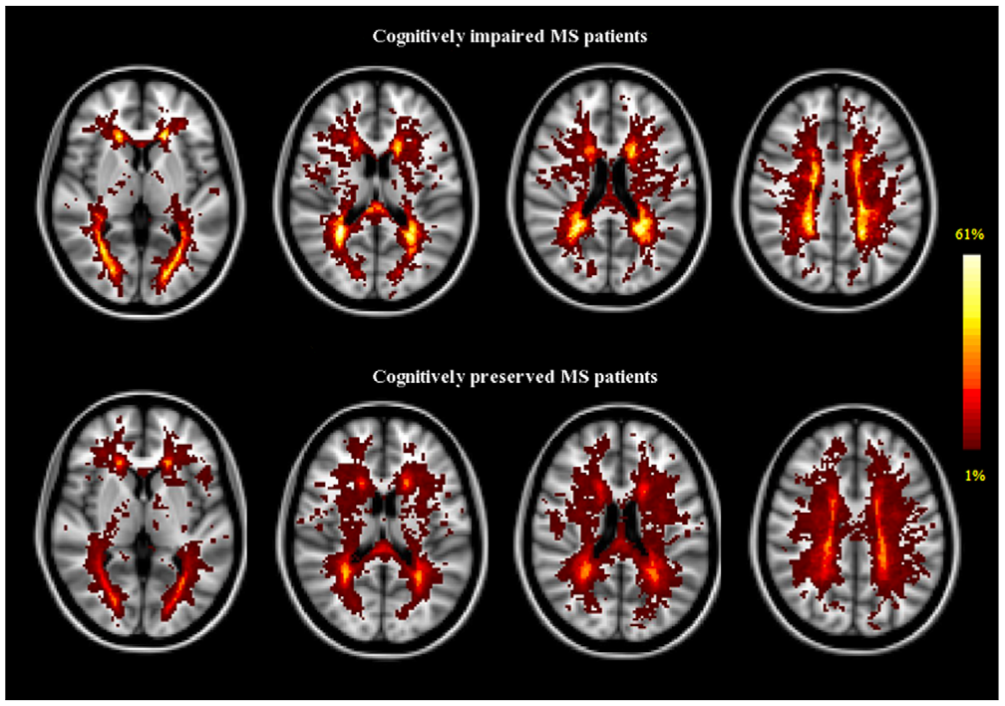
LPM in cognitively impaired and cognitively preserved patients with MS. T2-weighted lesion probability maps in cognitively impaired (upper panel, n = 36) and cognitively preserved (lower panel, n = 106) patients with MS. The color overlay created on top of the Montreal Neurological Institute standard brain shows the probability of each voxel containing a lesion in each patient group. The color bar denotes the probability range. The maximum local probability for lesions was higher in cognitively impaired patients (61% peak probability in the forceps major) than in cognitively preserved patients (37% peak probability in the posterior corona radiata). Images are shown in radiological convention.

The overall anatomical distribution of T2 lesions across the brain was similar in CP and CI patients (Figure 1). However, in the CI group T2 lesions tended to have a less widespread distribution despite their greater volume. The peak of lesion frequency was almost twice higher in CI (61% in the forceps major [FM]) than in CP patients (37% in the posterior corona radiata). Moreover the LPMi, which is a measure of the consistency of lesion pattern across subjects of a group, was higher in CI than in CP patients (16.5%±5.2% versus 9.4%±3%, p<0.001).

**Figure 2:**
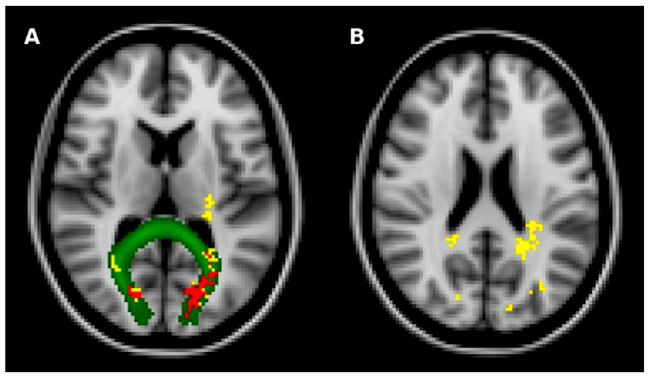
Clusters of high lesion frequency in cognitively impaired MS patients. Yellow shows the clusters of voxels where lesions, after controlling for age and sex, were more frequent (p<0.05, corrected) in cognitively impaired (n = 36) than in cognitively preserved (n = 106) patients with MS (forceps major in A and splenium of the corpus callosum in B). Red shows the clusters of voxels that also survived correction for T2-LV (forceps major, in A). Green represents the WM fiber tract (forceps major, in A) obtained from the FSL probabilistic tractography atlas. Background image is the MNI152 standard space image. Images are shown in radiological convention.

The voxelwise analysis confirmed that lesion frequency was higher in CI than in CP patients, with significant bilateral clusters in the FM and in the splenium of the corpus callosum (sCC) (p<0.05, corrected) (Figure 2, Table 3). After correcting also for T2-LV differences, only bilateral clusters in the FM were retained (p<0.05, corrected) (Figure 2).

**Table 3:** White matter regions of high lesion frequency in cognitively impaired MS patients.

WM region	Side	MNI (mm)			t-statistics	Cluster size	p-value
(local maxima)		X	Y	Z		(voxel number)	
Forceps major	L	−24	−70	8	5.28	788	0.001
	R	16	−82	16	4.64	135	0.004
Splenium of the corpus callosum	R	20	−44	24	4.81	42	0.012
	L	−20	−50	22	3.59	788	0.001

Local maxima within each significant cluster (p<0.05, corrected) where lesion frequency was higher in cognitively impaired than in cognitively preserved MS patients, after correcting for age and sex. After correcting also for T2-LV differences, only bilateral clusters in the FM were retained (p<0.05, corrected). See text for abbreviations.

#### Comparison among groups with different number of failed tests

To assess the relevance of the increasing number of failed tests on lesion location and frequency, a further analysis was performed considering three groups of MS patients: those with no test failed (n = 85 patients), those with one test failed (n = 21 patients) and patients with two or more tests failed (n = 36 patients). The FM, the sCC as well as the superior longitudinal fascicle (SLF) on the left hemisphere were the WM regions where the lesion frequency showed to be different among the three groups (p<0.05, corrected). In particular, patients with a high number of failed tests had higher lesion frequency in the FM and SLF (p<0.05, corrected) than patients who never failed at the BRB tests. After correcting also for T2-LV differences, a significant cluster was retained only in the FM. Patients with a high number of failed tests showed higher lesion frequency in the left sCC (p<0.05, corrected) than patients who failed one test at the BRB. This region survived correction also for T2-LV differences. By contrast, no significant differences in lesion frequency were found between patients who never failed and patients who failed one test at the BRB.

#### Correlation between BRB scores and lesion frequency

In general, the score of the different neuropsychological tests did not correlate with higher lesion frequency in specific brain regions, with the exception of the SDMT, where lower scores correlated with higher lesion frequency in the bilateral FM, sCC, left forceps minor (Fmin) and inferior fronto-occipital fascicle (IFOF) (p<0.05, corrected) (Table 4). In all these clusters, correlation between mean lesion signal intensity (i.e., the percentage of lesion voxels in each patient) and SDMT score was r =  -0.50, p<0.001. After correcting also for T2-LV, smaller clusters in the bilateral FM, sCC and left Fmin were retained (p<0.05, corrected). The mean signal intensity value within these clusters inversely correlated with SDMT scores (r = −0.55, p<0.001).

**Table 4:** White matter regions of higher lesion frequency with lower SDMT scores.

WM region	Side	MNI (mm)			t-statistics	Cluster size	p-value
(local maxima)		X	Y	Z		(voxel number)	
Forceps major	R	30	−52	12	4.39	480	0.001
	L	−16	−86	4	2.94	1220	0.001
Forceps minor	L	−16	30	2	4.38	46	0.005
Inferior fronto-occipital fascicle	R	34	−50	6	4.12	480	0.001
	L	−26	−70	0	3.53	1220	0.001
Splenium of the corpus callosum	R	18	−50	24	3.56	480	0.001
	L	−18	−52	22	3.24	1220	0.001

Local maxima within significant clusters (p<0.05, corrected) where lower SDMT scores correlated with higher T2 lesion frequency, after correcting for age and sex. After correcting also for T2-LV, smaller clusters in the bilateral FM, sCC and left Fmin were retained and correlation between the percentage of lesion voxels in these clusters and SDMT scores was r = −0.55, p<0.001. See text for abbreviations.

## Discussion

Given the very high detrimental impact of cognitive impairment on the daily life activities of a large proportion of MS patients, it is very important to know the mechanisms that can potentially contribute to its development and progression. In particular, it is still unclear from previous studies [23], [24], [30], [34], [35] whether there are significant differences in brain lesion location between CI and CP patients with MS, and consequently the relevance for cognitive impairment of the presence of a great lesion load in specific sites of the cerebral WM.

To provide an answer to this important issue, we exploited the potentials of the MRI-derived LPMs in assessing, at the voxel level, differences across populations in brain lesion spatial distribution and frequency and created LPMs of two groups of RRMS patients with and without cognitive impairment. We found that, despite CI patients had significantly greater LV than CP patients, their lesion patterns were more consistent across the brain, as also shown by their significantly higher LPMi. Indeed, the area with the highest probability of being lesion in CI patients was almost twice higher in CI than in CP, being localized to the FM (61%). Moreover, at voxelwise analysis, clusters of lesions in this area and in the sCC could distinguish CI patients from those who were CP (Figure 2). Overall, these results suggest that i) anatomic location of WM lesions is important in the development and progression of cognitive impairment, and ii) areas relevant for cognition lie mostly in the fibers crossing the CC.

Several MRI studies [4], [44] have shown that patients with greater lesion burden have significantly more cognitive impairment than those with less lesion burden. Some studies have also suggested that analysis of regional cerebral lesion load may help understanding the pattern and course of cognitive impairment in MS [23], [24], [30], [34], [35]. However, the overall relationship between cognitive impairment and lesion load was found to be weak-to-moderate and it is still unclear whether the presence of demyelinating lesions in a particular brain region is more relevant to cognitive impairment in MS. This has led to the notion that cognitive impairment cannot be adequately explained by the pathological features revealed by conventional MRI, which is somehow limited to the detection of macroscopic brain damage [4], [44]. In this study, however, by creating MRI-derived LPMs for each patient group, we showed that specific brain regions (i.e., FM and other areas of the CC) were more frequently classified as lesions in CI than in CP patients with MS, a finding that would have not been noted on MR images from single patients. Given the peculiar features of these regions, which are part of the commissural tracts connecting cortical and subcortical regions of both hemispheres, this result is particularly interesting. Indeed, this supports the early notion of a functional (multiple) disconnection between different GM structures, secondary to WM damage, as responsible for cognitive impairment in MS. Recent nonconventional MRI studies, by using diffusion tensor imaging (DTI) [20], [21], [45], [46], show a selective microstructural (i.e., non-lesional) damage in these regions of the CC and thus further support the hypothesis that a disconnection of these brain areas may play a role in the pathophysiology of cognitive impairment in MS.

Among the different cognitive tests, only the SDMT, which is a very sensitive measure of information processing speed, attention and working memory [47], showed a significant correlation with WM lesions. More specifically, low SDMT scores were associated with high lesion frequency in FM, Fmin, sCC and IFOF. The correlation with FM and sCC survived after correction for T2-LV. FM, which can be considered as the occipital radiation of the CC, has been associated with important cognitive domains such as attention, memory and executive functions [48], [49]. Indeed, the involvement of the FM in memory has been demonstrated by the presence of a fiber tract traveling between the sCC and hippocampus which connects to the fornix and the FM [50]. Moreover, a significant correlation was found between WM volume in the left FM and a factor of general cognition and mental ability in healthy old subjects [49]. Other recent studies have used LPM to assess correlations between a given cognitive test and specific brain regions in MS patients [23], [24], [30], [34], [35]. One study on CIS patients [24] showed that verbal learning performances were inversely associated with lesions in Broca's area, right frontal lobe and sCC and that spatial learning performances were inversely correlated with lesions in the deep WM, whereas it did not find significant correlations between lesion location and performance in tasks exploring processing speed and executive functions. Another study [23], including patients with different MS subtypes, found significant correlation of WLG with lesions in the left SLF and, when data were not corrected for multiple comparisons, could also show correlation of SDMT with lesions in the left frontal lobe. Beside differences due to patient populations or technical approaches, it is possible that measures of information processing speed, attention and working memory such as SDMT might be more sensitive to pronounced tissue damage (i.e., high lesion load) as the one reported in our study [51]. However, a very recent DTI study [52] showed very close correlation between low SDMT scores and mostly non-lesional brain regions such as CC, sagittal stratum and posterior thalamic radiation (including optic radiation), suggesting that microstructural damage may exist in these regions even before macroscopic lesions become evident. Finally, the close relationship of SDMT, which is a measure of visual processing speed, with tissue damage in regions containing fiber tracts connecting to the occipital cortex (i.e., FM and optic radiation), as found in our LPM study and in a previous DTI work [53], might provide a biological explanation of the visual perceptual deficits occurring in MS patients with cognitive impairment.

Voxel-based analyses such as those performed here have strengths and limitations. A strength that should be considered is the methodological approach used to create the LPMs. We used here the nonlinear registration to align patients' brain, which diminishes the potential bias coming from errors in registration and greatly improves the alignment upon visual inspection. Moreover, we carried out analyses by using nonparametric permutation tests, which are particularly useful when the study groups are composed of unequal numbers of subjects [42], as the data presented here. Finally, as most recent studies [23], [31], we applied for data analysis TFCE, which uses the spatial information inherent in the data to calculate statistical maps, thus not requiring arbitrary presmoothing of images and not depending on an arbitrary initial cluster-forming threshold [43], [52]. By contrast, a limitation of the study lies in the selective use of WM T2 lesions, without assessing, for example, differences in cortical LPM [28] or cortical atrophy [11]. On a different perspective, another limitation of our study might be represented by the fact that fatigue and depression scales (such as FSS and MADRS) were not acquired. However, we did not include in our study subjects with major mood disorders or on treatment with psychoactive drugs.

## Conclusions

It is well know that the cognitive impairment in MS has complex and multifactorial causes, which cannot be adequately and exclusively explained by pathological features captured by an unspecific measurement such as T2 lesions. The results reported here, however, support the notion that focal damage in specific brain WM regions (i.e., commissural fibers) is particularly relevant to cognition. This might contribute to improve the knowledge on the pathophysiology of cognitive impairment in MS, potentially leading to the development of better outcome measures and targets for new treatment strategies in this disabling condition.
